# Effects of iron supplementation and ET-18-OCH3 on MDA-MB 231 breast carcinomas in nude mice consuming a fish oil diet.

**DOI:** 10.1038/bjc.1997.389

**Published:** 1997

**Authors:** W. E. Hardman, C. J. Barnes, C. W. Knight, I. L. Cameron

**Affiliations:** Department of Cellular and Structural Biology, The University of Texas Health Science Center, San Antonio 78284-7762, USA.

## Abstract

**Images:**


British Journal of Cancer (1997) 76(3), 347–354
© 1997 Cancer Research Campaign

### Effects of iron supplementation and ET-18-OCH$_3$ on MDA-MB 231 breast carcinomas in nude mice consuming a fish oil diet

**WE Hardman, CJ Barnes, CW Knight and IL Cameron**

Department of Cellular and Structural Biology, The University of Texas Health Science Center, San Antonio, TX, USA

**Summary** Lipid peroxidation products can be cytotoxic. Our objectives were (1) to use two pro-oxidants (iron and a pro-oxidative drug) to selectively increase lipid peroxidation in the implanted human breast tumours of mice consuming fish oil and (2) to kill the cancer cells without harming normal host tissues. The theoretical basis for selective cytotoxicity is that normal cells are better able to handle oxidative stress than cancer cells. Male athymic nude mice, consuming an AIN-76 diet, were injected s.c. with MDA-MB 231 human breast carcinoma cells. Three weeks later, all mice had palpable tumours, 3–10 mm in diameter, and diets were changed to modified AIN-76 diets containing 19% menhaden fish oil and 1% corn oil with or without supplemental 0.3% ferric citrate. After 2 weeks, half of the mice on each diet (19% fish oil with or without supplemental ferric citrate) were injected (three times per week for 2 weeks) with the ether–lipid drug edelfosine (ET-18-OCH$_3$). The concentration of lipid peroxidation products in tumours (as measured by thiobarbituric acid-reactive substances, TBARS) was significantly increased by both ferric citrate and ET-18-OCH$_3$. The TBARS in livers were not increased, nor was there evidence of other harmful side-effects to the host mice. The addition of iron enhanced tumour cell death whereas ET-18-OCH$_3$ suppressed tumour cell mitosis. The use of iron supplementation combined with ET-18-OCH$_3$ resulted in the slowest growth rate, lowest mitotic index, highest level of lipid peroxidation products and increased the cytotoxic index in tumours without detectable harm to the host. That iron supplementation increased tumour suppression beyond that expected from the increase in the concentration of TBARS in the tumour merits further investigation.

**Keywords:** lipid peroxidation; breast cancer; fish oil; edelfosine (ET-18-OCH$_3$)

Previous studies have revealed that a high level of fish oil in the diet of nude mice can retard the growth or cause regression of implanted human breast cancers (Borgeson et al, 1989; Gonzalez et al, 1991; 1993). The addition of iron to the diet of mice consuming fish oil further suppressed tumour growth (Gonzalez et al, 1991). One proposed mechanism for the tumour regression under these dietary conditions is that cancer cell death results from an increase in the products of lipid peroxidation following consumption of fish oil (Gonzalez et al, 1991). When fish oil is consumed, the highly polyunsaturated fatty acids (HPUFAs) from the fish oil are rapidly incorporated into the cellular membranes. Lipid peroxidation products are formed when membrane polyunsaturated fatty acids (PUFAs) undergo oxidative damage; this oxidative damage can be enhanced if iron is available to catalyse the production of reactive oxygen species. Free radicals formed during lipid peroxidation may physically damage cellular lipid membranes, proteins and DNA (Masotti et al, 1988) and/or induce cell death by apoptosis (McConkey and Orrenius, 1994) or other forms of cell death. In addition, some of the products of lipid peroxidation (e.g. malondialdehyde, 4-hydroxy-2-nonenal and 4-hydroxy-2-hexenal) are directly toxic to the cells in relatively low concentrations (Esterbauer et al, 1991). Increased lipid peroxidation has been thought to kill cancer cells preferentially because the activities of cellular antioxidants that protect normal cells against oxidative damage (i.e. catalase and glutathione peroxidase) may be decreased in cancer cells (Corrocher et al, 1986). Thus cancer cells are less able than normal cells to inactivate the oxygen radicals formed as a consequence of lipid peroxidation and are therefore less able to survive increased lipid peroxidation.

*Received 1 December 1996*
*Revised 1 December 1996*
*Accepted 17 January 1997*

*Correspondence to:* WE Hardman, Department of Cellular and Structural Biology, The University of Texas Health Science Center at San Antonio, 7703 Floyd Curl Dr, San Antonio, Texas 78284-7762, USA

Results of previous studies using either cultured cells (examples: Begin et al, 1986; 1988; Canuto et al, 1991; Grammatikos et al, 1994; Maehle et al, 1995; Padma and Das, 1996) or whole animals (examples: Borgeson et al, 1989; Pritchard et al, 1989; Gonzalez et al, 1991; 1993) have indicated that an increased exposure to the types of HPUFAs found in fish oil, especially eicosapentanoic acid, kills a variety of cancer cells without killing normal cells, and that addition of an iron-containing compound may increase cell death. However, the question still remains as to whether the most important cause of tumour regression and cell death is: (1) induction of lipid peroxidation; (2) alteration of eicosanoid production; (3) some other mechanisms associated with the alteration in membrane fatty acids or (4) the addition of iron to the diet or culture media.

We reasoned that if lipid peroxidative damage was the main cause of tumour regression then systematically increasing lipid peroxidation in tumours should increase tumour regression and tumour cell death. To test this idea: (1) tumour-bearing nude mice were fed a diet containing fish oil (which is rich in HPUFAs) to provide the substrate for lipid peroxidation in the cancer cells; (2) the fish oil diet was supplemented with a pro-oxidant, ferric citrate, at 0.3% (w/w) to enhance lipid peroxidation; (3) one half of the mice consuming fish oil with supplemental ferric citrate and one half of the mice consuming fish oil without supplemental ferric citrate were treated with the ether–lipid drug edelfosine ($ET\text{-}18\text{-}OCH_3$). $ET\text{-}18\text{-}OCH_3$ is readily incorporated into cellular membranes and is hypothesized to kill cancer cells by inducing lipid peroxidative damage to the plasma membrane (Wagner et al, 1992). In support of this mechanism of action for $ET\text{-}18\text{-}OCH_3$, it has been shown in cell culture studies that: (1) lipid peroxidation was increased by addition of $ET\text{-}18\text{-}OCH_3$ to cell cultures that contained fish oil and ferric citrate and (2) cytotoxicity correlated with the increase in lipid peroxidation when oxidative co-factors (iron and ascorbic acid) were present (Wagner et al, 1992). Thus, treatment of the host mouse with $ET\text{-}18\text{-}OCH_3$ in addition to the fish oil and ferric citrate in the diet was expected to increase lipid peroxidation in the tumour.

Briefly, the results of this study indicate that lipid peroxidation was significantly increased by supplementing a 19% fish oil diet with ferric citrate and by periodic injections of $ET\text{-}18\text{-}OCH_3$. However, iron supplementation to a fish oil diet appears to make an additional contribution to tumour suppression beyond that attributed to the ability of iron to increase the concentration of TBARS in the tumour. The mechanism by which iron supplementation makes this additional contribution to tumour growth suppression has not been elucidated.

### MATERIALS AND METHODS

### Tumour cells

MDA-MB 231 human breast carcinoma cells (American Type Culture Collection, Rockville, MD, USA) were cultured for injection into the tumour-bearing mice. The culture medium was an enriched L15:SMEM base media supplemented with other factors as described previously (Moyer and Aust, 1984).

### Animals

Thirty-six male athymic nude mice (nu/nu, Harlan Sprague Dawley, Madison, WI, USA), 6 weeks old, were used in this study. The mice were housed under aseptic conditions (positive pressure, designated nude mouse room, sterilized cages with microisolator tops, sterile bedding and water) in a temperature- (24°C) and light-controlled (12 h $day^{-1}$) room. All mouse handling was carried out under a laminar flow hood. All animal use and handling was approved by our Institutional Animal Care and Use Committee before commencing the experiment. The animal care facilities are accredited by the American Association for the Accreditation of Laboratory Animal Care.

### Experimental design

Cultured MDA-MB 231 cells were harvested, rinsed then suspended in serum-free, L15:SMEM culture medium. Cells in suspension were counted using a Coulter cell counter and the cell count was adjusted to $20 \times 10^6$ cells $ml^{-1}$. The suspension was kept well mixed during the time of injection. Cancer cells ($10^6$ cells in 0.05 ml of serum-free culture medium) were injected subcutaneously (s.c.) over each scapula on the upper back of the nude mice. The MDA-MB 231 human breast carcinoma forms tumours after s.c. injection into male nude mice and does not require supplemented hormones for growth. Male mice were used to prevent possible cyclic hormonal influences on tumour growth. Palpable, measurable tumours were found beginning 10 days after the cells were implanted and by 3 weeks the tumours had grown at 99% of the injection sites. The lengths and widths of palpable tumours were measured three times weekly with Vernier calipers during the entire experiment to establish growth curves of each tumour before and after the dietary and $ET\text{-}18\text{-}OCH_3$ treatments. All mice were weighed weekly.

Mice were fed a standard AIN-76 purified diet (Table 1) from receipt until 3 weeks after transplantation of the human breast carcinoma cells. This allowed the carcinoma cells to become established as growing tumours in the host mice before the onset of the experimental dietary treatments. The cancer-bearing mice were then divided into two groups (18 mice each). All mice had free access to a modified AIN-76 purified diet (Table 1), which contained 19% fish oil. The mineral mix incorporated into the basal diet contained 0.6% ferric citrate (16–17% Fe), thus the basal diet contained 0.02 g ferric citrate 100 $g^{-1}$ dry weight of food. Supplemental ferric citrate was added to the diet of one group of 18 mice at a rate of 0.3 g 100 $g^{-1}$ dry weight of food. On a caloric basis, the diets were balanced to the standard AIN-76 diet for protein, minerals, vitamins and fibre; the adjustment for the calories in the 19% fish oil diets was made by subtracting sucrose calories. One per cent corn oil was included in the fish oil diets to provide sufficient essential linoleic acid for mouse and tumour growth.

The diets were prepared weekly, individual daily portions for each cage were packaged and the packages were stored in sealed containers at –20°C to suppress spontaneous lipid peroxidation.

**Table 1** Composition of the diet by weight per cent (g per 100 g of food)

| Ingredient | 5% Corn oil | 19% Menhaden oil/1% corn oil |
|---|---|---|
| Corn oil | 5.0 | 1.0 |
| Menhaden oil | – | 19.0 |
| Sugar | 50.0 | 27.9 |
| Casein | 20.0 | 23.2 |
| Cornstarch | 15.0 | 17.4 |
| AIN-76 vitamin mix[a] | 1.0 | 1.15 |
| AIN-76 mineral mix[a] | 3.5 | 4.06 |
| Choline bitartrate | 0.2 | 0.23 |
| DL-methionine | 0.3 | 0.35 |
| Cellulose | 5.0 | 5.8 |
| Total | 100.0 | 100.1 |
| Composition of the diets by % calories[b] | | |
| Protein | 20.7 | 20.6 |
| Carbohydrate[c] | 67.5 | 40.1 |
| Fat | 11.6 | 39.3 |
| Energy content of each diet kcal $g^{-1}$ | 3.85 | 4.52 |

[a]α-Tocopherol is 0.02 g 100 $g^{-1}$ and ferric citrate (16–17% $Fe^{3+}$) is 0.02 g 100 $g^{-1}$ of the basal diet. [b]Caloric content is calculated at 4 kcal $g^{-1}$ for protein and carbohydrate and 9 kcal $g^{-1}$ for fat. The diet that included a pro-oxidant (iron) had 0.3 g 100 $g^{-1}$ of ferric citrate (16–17% $Fe^{3+}$) added to the 19% Menhaden oil diet. [c]The percentage of calories from carbohydrate include the calories from sucrose, cornstarch and sucrose in the vitamin and mineral mix. Diet components and chemicals: purified high-nitrogen casein, pure corn starch, Alphacel (non-nutritive bulk cellulose) AIN-76 vitamin mixture, AIN-76 mineral mixture and choline bitartrate (99% pure) was obtained from ICN Nutritional Biochemicals, Cleveland, OH, USA. Imperial brand (Sugarland, TX, USA) extra-fine pure cane sugar and 100% pure corn oil (Wesson) was purchased locally. DL methionine (cell culture, MW 149.2), menhaden fish oil and ferric citrate was purchased from Sigma, St Louis, MO, USA.

© Cancer Research Campaign 1997

The food was replaced every day and the weight of food consumed per cage per day was recorded. Gonzalez et al (1992) reported that there was not a significant increase in peroxidation products in 19% fish oil diets when food was stored at –20°C; however, there was significant peroxidation after 24 h at room temperature. As the diets were not sterilized, antibiotics (bacitracin, streptomycin and neomycin, at 1 g each per l) were added to the drinking water to prevent infection.

The mice were maintained on the fish oil diets for 2 weeks to allow HPUFA substitution of the membrane fatty acids before beginning treatment with ET-18-$OCH_3$ (edelfosine, Medmark Pharma). ET-18-$OCH_3$ was selected to further increase lipid peroxidation based on the conclusion that the mechanism of action of ET-18-$OCH_3$ was through induction of lipid peroxidation at the cellular membranes (Wagner et al, 1992). A non-lethal dose of ET-18-$OCH_3$, as reported in a previous study (Leder et al, 1987), was selected. Half of the mice in each dietary group received ET-18-$OCH_3$, at a dose of 10 mg drug $kg^{-1}$ body weight injected s.c. on the left rear flank three times per week for 2 weeks. The mice received either 0.1 ml of the drug solution (30 mg of the drug was dissolved in 10 ml of sterile 0.9% Sodium chloride) or of the saline (controls) for each 30 g body weight.

### Tissue collection and preparation

The experiment was terminated 2 weeks after the initiation of ET-18-$OCH_3$. The mice were anaesthetized using a ketamine/S.A. rompun mixture (0.2 ml 25 $g^{-1}$ weight, i.m.) prepared by our Laboratory Animal Resources veterinarian, then terminated by cervical dislocation. Immediately after cervical dislocation, a blood sample (about 0.5–1 ml) for serum analyses was obtained by cardiac puncture. Subcutaneously growing primary human breast carcinomas were excised, weighed, the dimensions measured and their volumes calculated by using the formula for the volume of a prolate spheroid ($V = \pi/6 \times$ length $\times$ width $\times$ depth). Terminal volume of the excised tumours correlated significantly ($P < 0.001$) with the terminal volume of tumours measured in the live mice. This indicated that the in situ tumour volume as estimated in live mice was reliable. The lungs, liver and spleen were removed then inspected for tumours and gross pathology. Each specimen of primary cancer or host organ was rinsed with ice-cold 0.9% sodium chloride, the excess saline was blotted and the specimen was weighed. A piece of each specimen was fixed in 10% neutral buffered formalin for 2 h then transferred to 70% ethanol for later processing to histological slides. The remainder of each cancer or organ was placed in 0.9% sodium chloride, flash frozen in liquid nitrogen and stored at –90°C until further assays could be performed.

**Figure 1** Photomicrograph of a 4-µm-thick section of a human MDA-MB 231 breast carcinoma grown in a nude mouse. Examples of a dying cell (arrowhead) and of a cell in mitosis (arrow) are indicated (haemotoxylin–eosin-stained, magnification, ×1000)

### Tissue analyses

At a later date, frozen tumours and normal organs were thawed and homogenized individually at 4°C using a Polytron homogenizer. An aliquot of the whole specimen homogenate was reserved for analysis of total protein content. The total protein content of the tissue homogenate of the tumour and of the host liver was measured by the method of Bradford (1976) using the Bio-Rad protein assay (micro-method).

The remainder of the homogenate was used in the TBARS assays to estimate lipid peroxidation. Malondialdehyde and other products of lipid peroxidation can be estimated spectrophotometrically at 535 nm after reaction with thiobarbituric acid to obtain an index for lipid peroxidation (Esterbauer et al, 1991). We realize that TBARS do not measure all products of lipid peroxidation and that there may be minor interference by other substances (sugars, amino acids, etc.); however, this simple inexpensive test does provide a good estimate of changes in overall lipid peroxidation of tissues. The absorbance values obtained were compared with a standard curve of known concentrations of malondialdehyde and normalized by protein content of the specimen. The results are reported as nmol of TBARS per mg of protein.

The cytotoxic and mitotic indices were obtained from coded haematoxylin–eosin-stained histological sections of the tumour (see Figure 1). All scoring of the coded sections was done by one individual, CWK, without knowledge of the treatment given to the mouse. Cell death was determined by morphological indicators of either apoptosis (marginalization of chromatin to the nuclear envelope, nuclear fragmentation, cytoplasmic shrinkage) or necrosis (pyknotic nucleus, eosinophilic cytoplasm, no cell shrinkage). Mitosis was determined by the presence of mitotic figures. At least 1000 contiguous cells in non-necrotic sections of each tumour were counted. The cytotoxic and the mitotic indices were expressed as the percentage of cells that were undergoing cell death or that were in mitosis respectively.

Sizes of the axillary lymph nodes were recorded when enlargement was detected by palpation. At necropsy, metastatic tumours were found in the axillary lymph nodes of almost all of the mice. These were excised and the tumour cells were identified by histology to be of epithelial origin; however, no further testing has been completed. The lymph node data were not adequate to generate growth curves of lymph node tumours because of the large variation in the ratio of host–tumour tissue in the lymph node.

© Cancer Research Campaign 1997

Table 2 Caloric consumption, terminal body weights and terminal liver or spleen weights of groups of mice that consumed different dietary modifications for 4 weeks. Half of the mice on each diet received ET-18-$OCH_3$ for 2 weeks before killing ($n$ = 8–10 mice per group)

| Diet | With (+) or without (–) ET-18-$OCH_3$ | Calories (kcal per mouse day$^{-1}$) | Body weight (g per mouse ± s.e.) | Liver (g per mouse + s.e.) | Spleen (g per mouse ± s.e.) |
|---|---|---|---|---|---|
| 19% Fish oil | – | 21.35 ± 1.14 | 33.3 ± 1.48 | 1.70 ± 0.13 | 0.20 ± 0.01 |
| 19% Fish oil | + | 21.24 ± 1.07 | 38.7 ± 0.69 | 1.86 ± 0.09 | 0.22 ± 0.01 |
| 19% Fish oil + ferric citrate | – | 18.66 ± 0.93 | 37.0 ± 1.64 | 1.80 ± 0.04 | 0.23 ± 0.02 |
| 19% Fish oil + ferric citrate | + | 17.66 ± 1.23 | 35.3 ± 0.92 | 1.66 ± 0.08 | 0.21 ± 0.01 |

ANOVA showed that there were no significant differences in the mean caloric consumption, mean body, liver or spleen weights of the groups of mice with different dietary modifications or ET-18-$OCH_3$.

Table 3 Mean thiobarbituric acid reactive substances (TBARS in nmol TBARS mg$^{-1}$ protein ± s.e.) in the liver or in the human breast carcinoma of groups of mice consuming different dietary modifications with and without ET-18-$OCH_3$ treatment injected s.c. at 10 mg kg$^{-1}$ body weight three times per week for 2 weeks before killing ($n$ = number of mice)

| Diet | No ET-18-$OCH_3$ | *n* | With ET-18-$OCH_3$ | *n* | Row means |
|---|---|---|---|---|---|
| *TBARS in liver*[a] | | | | | |
| 19% Fish oil | 0.25 ± 0.056 | 7 | 0.30 ± 0.054 | 7 | 0.28 ± 0.038 |
| 19% Fish oil + ferric citrate | 0.17 ± 0.061 | 5 | 0.34 ± 0.050 | 6 | 0.26 ± 0.045 |
| Column means | 0.21 ± 0.042 | | 0.32 ± 0.036 | | |
| *TBARS in tumour*[b,c] | | | | | |
| 19% Fish oil | 0.11 ± 0.050 | 5 | 0.81 ± 0.176 | 9 | 0.56 ± 0.144 |
| 19% Fish oil + ferric citrate | 0.54 ± 0.243 | 3 | 1.15 ± 0.233[c] | 7 | 0.96 ± 0.183 |
| Column means | 0.27 ± 0.131 | | 0.96 ± 0.133 | | |

[a]Two-way ANOVA of TBARS in the host liver showed that there was no significant difference due to the ET-18-$OCH_3$ (column means, $P < 0.08$) or due to the diet (row means, $P = 0.87$). There were no significant interactions between the diet and the ET-18-$OCH_3$. [b]Two-way ANOVA of TBARS in the implanted human breast carcinoma showed that there was a significant increase because of the ET-18-$OCH_3$ (column means, $P < 0.004$) and due to ferric citrate (row means, $P = 0.05$). There were no significant interactions between the diet and the ET-18-$OCH_3$. [c]One-way ANOVA showed that the content of TBARS in the tumours of the group of mice that consumed supplemental ferric citrate and received ET-18-$OCH_3$ was significantly greater than that in the tumours of the group that consumed the fish oil diet.

Diagnostic serum enzyme levels were evaluated to determine whether or not there were side-effects (damage to other tissues of the host mouse) because of consumption of any of the diets. The enzymes analysed were (an elevation of a specific enzyme activity indicates damage to the tissues listed in parenthesis; Davidson and Henry, 1974): alkaline phosphatase (bone, liver); serum glutamic oxaloacetic transaminase (heart, liver, skeletal muscle, kidney); serum glutamic pyruvic transaminase (liver); lactic dehydrogenase (increased with a variety of tissue damage but especially liver damage); amylase (pancreas); creatine phosphokinase (thyroid, brain, lung, heart). These analyses are routine clinical chemistry analyses and were performed by the Clinical Pathology Laboratory at University Hospital (San Antonio, TX, USA). Appropriate controls were used for all tests to establish the linearity of the procedures. The laboratory is certified by the College of American Pathologists.

### Statistical analyses

All samples were coded before analyses to minimize bias. SAS statistical software was used for statistical analyses. Tests for normality were performed on each data set. Two-way analysis of variance (ANOVA) followed by one-way ANOVA and Student–Newman–Keuls multiple range tests were used to determine if there were statistically significant ($P \leq 0.05$) differences between the groups because of the ferric citrate, the ET-18-$OCH_3$ or interactions between the ferric citrate diet and ET-18-$OCH_3$.

Linear regression analysis was used to determine if the increase in the mean tumour volume over the time of the experiment showed a significant linear fit. Slope analysis for differences between the regression of mean tumour volume per group was performed by SAS using the general linear model procedure to generate an ANOVA followed by a $T$-test between each pair of lines against the null hypothesis that there was no difference between the slopes. A $P$-value $\leq 0.05$ was used to indicate that there was a significant difference between slopes of the regression lines, and thus that the tumour growth rates represented by the slopes were significantly different.

### RESULTS

### Dietary modifications and ET-18-$OCH_3$ on the tumour-bearing host mouse

ANOVA showed that there were no significant differences between experimental groups of mice in: (1) body weight (Table 2); (2) daily caloric consumption (Table 2) or (3) the weights of liver or spleen (Table 2). Routine clinical chemistry analyses were performed on individual mouse serums for the amount of the enzymes; alkaline phosphatase, serum glutamic oxaloacetic

© Cancer Research Campaign 1997

**Figure 2** Growth rate of MDA-MB231 human breast carcinoma in nude mice. Mice with tumours were divided into groups and fed modified AIN-76 diets containing 19% fish oil with and without ferric citrate. After 2 weeks on the fish oil diet, half of the mice on each diet were injected with ET-18-OCH three times per week for 2 weeks. The diets and treatments were: 1, fish oil; 2, fish oil with ET-18-$OCH_3$; 3, fish oil with ferric citrate; 4, fish oil, ferric citrate and ET-18-$OCH_3$. See Table 4 for the summary of statistical analyses of these data transaminase, serum glutamic pyruvic transaminase, lactic dehydrogenase, amylase, creatine phosphokinase. ANOVA showed no significant differences between groups in the amounts of any of the six serum enzymes (data not shown). There were no apparent differences between groups in the gross pathology or histology of the liver, lung or spleen. Taken together, these results indicate that neither the supplementation with ferric citrate nor ET-18-$OCH_3$ treatment had a detectable or significant damaging effect on the liver, bone, spleen, heart or pancreas or in any noticeable way altered the tissues or the overall health status of the mice.

Likewise, analyses of the concentration of TBARSs (an indication of lipid peroxidation) in the liver of the host mice (Table 3) showed no significant main effects because of either the dietary modification or ET-18-$OCH_3$ treatment.

Supplementation of the 19% fish oil diet with 0.3% ferric citrate did not significantly increase the serum iron level above that of the mice given 0.02% ferric citrate in their food. The mean serum iron level of the group of mice fed 19% fish oil with 0.02% dietary ferric citrate was 161.6 ± 8.8 mg $dl^{-1}$ (mean ± s.e.); the mean serum iron level of the group of mice fed 19% fish oil with supplemental 0.3% ferric citrate was 173.0 ± 10.9 mg $dl^{-1}$. ANOVA revealed that these serum iron levels were not significantly different.

**Table 4** Mean growth rates of the implanted MDA-MB 231 human breast carcinomas of groups of mice that consumed different diets for 4 weeks. Half of the mice on each diet group received ET-18-$OCH_3$ for 2 weeks before killing ($n$ = 8–10 mice per group)

| Group | Diet[a] | With (+) or without (–) ET-18-$OCH_3$ | Growth rate[b] ($mm^3$ $day^{-1}$ ± s.e.) | Correlation to linear regression[c] ($r$) | Growth rate is significantly different[d] from all groups except |
|---|---|---|---|---|---|
| 1 | 19% Fish oil | – | 9.64 ± 1.2 | 0.97 | |
| 2 | 19% Fish oil | + | 3.97 ± 0.5 | 0.95 | |
| 3 | 19% Fish oil + ferric citrate | – | 0.43 ± 0.4 | 0.20 | 4 |
| 4 | 19% Fish oil + ferric citrate | + | 0.35 ± 0.3 | 0.30 | 3 |

[a]The composition of the diets is listed in Table 1. [b]Slope of the best fit linear regression line ± s.e. of that slope. [c]The slope of the line of groups 1 and 2 shows a significant ($P < 0.05$), positive linear fit by linear regression analysis. The slope of the line of groups 3 and 4 is not significantly different from 0. [d]The growth rates (slopes) were compared by SAS using a general linear model to generate an analyses of variance followed by a $T$-test against the null hypothesis that there was no difference between the slopes of each pair of lines. $P \leq 0.05$ was used to indicate significant differences.

**Table 5** Mitotic and cytotoxic indices (± s.e.) in the implanted MDA-MB 231 tumours from groups of mice that consumed different dietary modifications for 4 weeks either without or with ET-18-$OCH_3$ treatment for 2 weeks before killing ($n$ = number of mice scored for each mean)

| Diet | No ET-18-$OCH_3$ mean + s.e. ($n$) | With ET-18-$OCH_3$ mean + s.e. ($n$) | Row means |
|---|---|---|---|
| *Mitotic index (%)* | | | |
| 19% Fish oil | 2.17 ± 0.69 (3) | 1.16 ± 0.09 (9) | 1.41 ± 0.21 |
| 19% Fish oil + ferric citrate | 1.72 ± 0.54 (5) | 1.15 ± 0.05 (9) | 1.36 ± 0.16 |
| Column means | 1.90[a] ± 0.34 | 1.16 ± 0.05 | |
| *Cytotoxic index* (%) | | | |
| 19% Fish oil | 3.86 ± 0.91 (3) | 5.84 ± 0.81 (9) | 5.34 ± 0.68 |
| 19% Fish oil + ferric citrate | 9.85 ± 2.74 (5) | 7.19 ± 1.29 (9) | 8.14 ± 1.14[b] |
| Column means | 7.6 ± 1.71 | 6.5 ± 0.76 | |

[a]Two-way ANOVA revealed a significant difference in the mitotic index in the tumour because of the ET-18-$OCH_3$ treatment (column means, $P$ = 0.006). there was no significant difference in the mitotic index as a result of the diets (row means, $P$ = 0.80). [b]Two-way ANOVA revealed a marginally significant increase in the cytotoxic index in the tumour as a result of the consumption of ferric citrate (row means, $P$ = 0.053). There was no significant difference in the cytotoxic index due to the ET-18-$OCH_3$ treatment (column means, $P$ = 0.62).

### Dietary modification and ET-18-$OCH_3$ on the implanted human breast carcinoma

Two-way ANOVA (Table 3) of the concentration of TBARSs in the implanted human breast carcinoma revealed that there was a significant increase in TBARS because of the ferric citrate supplementation ($P = 0.05$) and ET-18-$OCH_3$ ($P < 0.004$).

The mean tumour volume of each group was normalized to a volume of 0 at the beginning of the fish oil diets. This was accomplished by subtracting the mean tumour volume at the beginning of the fish oil from the tumour volume at that time and at each subsequent measurement. The normalized mean daily tumour volume for each dietary group plotted as a function of the number of days after initiation of the fish oil diet is presented in Figure 2. Least-squares linear regression analyses of tumour volume vs time showed that the tumour growth rate was significantly less because of addition of ferric citrate or because of treatment with ET-18-$OCH_3$ (summarized in Table 4). Figure 2 illustrates that there was not a significant increase in the mean tumour volume of mice that consumed high fish oil plus ferric citrate (with or without ET-18-$OCH_3$), thus the mean tumour growth of these two groups was nil.

The mitotic and cytotoxic indices (per cent of cells in mitosis or per cent of dying cells respectively) were determined from 4-μm-thick histological sections of non-necrotic areas of the tumour. Two-way ANOVA of the mitotic index (Table 5, row means) revealed that the ferric citrate supplementation to the diet of the mice did not significantly alter the mitotic index in the tumours. However, ET-18-$OCH_3$ treatment did significantly ($P < 0.05$) decrease the mitotic index (Table 5, column means) in the tumours of mice that received the ET-18-$OCH_3$ compared with the mice that did not receive ET-18-$OCH_3$.

Two-way ANOVA of the cytotoxic index indicated that there was a marginally significant increase ($P = 0.053$) in the percentage of dying cells because of ferric citrate supplementation. Specifically, the results of one-way ANOVA demonstrated that consumption of the diet containing high fish oil plus ferric citrate significantly increased the cytotoxic index in the tumour (row means). There was not a significant difference in the cytotoxic index because of the ET-18-$OCH_3$ treatment (column means).

When interpreting the mitotic, cytotoxic and TBARS values, it should be noted that some tumours regressed because of the diet and/or ET-18-$OCH_3$ treatment and were either undetectable or too small to analyse at the time of necropsy. Thus, for these tumours, there was insufficient or no tumour remaining to analyse for mitotic index, for cytotoxic index or for TBARS content. The lack of measured values on tumours that regressed to the greatest extent influences the ability to detect statistically significant changes in mitotic index, cytotoxic index or TBARS because of the diet and/or the ET-18-$OCH_3$.

**Figure 3** The linear regression analyses (± 95% confidence interval) of concentration of lipid peroxidation products (thiobarbituric acid-reactive substances, TBARS) in MDA-MB 231 human breast carcinomas grown in nude mice against the mean tumour growth rate ($mm^3$ $day^{-1}$). Diets and treatments are indicated. Error bars indicate s.e.m. for the growth rate of the group (see Table 4 for data), $n = 8–10$ mice per group. Dashed lines indicate the 95% confidence interval for the regression line with the fish oil plus supplemental ferric citrate group excluded from the regression analyses as it is outside the 95% confidence interval. The slope of the regression line = –8.80, $P$ for slope = 0.05

### DISCUSSION

The main purpose of this study was to evaluate whether increasing lipid peroxidation, as measured by TBARS, in the tumour would correlate with decreased growth rates in implanted human breast carcinomas and to determine if there was detectable harm to the tumour-bearing host mice.

### Comparisons with similar past research

The research results in this report are in general agreement with other published findings and we add several new findings. Our results confirm those of earlier reports (Gonzalez et al, 1991, 1993) that supplementation of the high fish oil diet with a pro-oxidant, ferric citrate, suppresses breast cancer growth and increases lipid peroxidation products in human breast cancer growing in nude mice. Our original findings are as follows.

1. The significant accumulation of lipid peroxidation products in the breast cancer cells was not accompanied by a significant increase in lipid peroxidation products in the host liver. This indicates that there was selectivity of the intervention between the cancer cells and a representative normal host cell population.
2. Addition of the ether–lipid drug ET-18-$OCH_3$ increased lipid peroxidation in the tumour and the increased lipid peroxidation generally correlated with a decreased tumour growth rate. However, dietary supplementation with iron in the form of ferric citrate caused a greater suppression in the growth rate than would be expected from the measured increase in lipid peroxidation.
3. Addition of iron to the fish oil diet caused a significant increase in the cytotoxic index (cell death) of the tumour without a decrease in the mitotic index (rate of cell division), whereas the addition of ET-18-$OCH_3$ to the fish oil diet caused a significant decrease in the mitotic index of the tumour without a significant increase in the cytotoxic index. It therefore seems that iron supplementation and ET-18-$OCH_3$ suppress tumour growth via different mechanisms. The exact mechanisms involved remain to be elucidated.

### Assessment of the dietary modifications and ET-18-$OCH_3$ on the host

It was thought important to look for harmful side-effects of the treatments on the host, as detrimental side-effects would influence

© Cancer Research Campaign 1997

the applicability of the same or a similar treatment to humans with cancer. The diagnostic serum enzymes tested are commonly used clinically to indicate organ damage or disease. In addition, histological sections of the liver, lungs and spleen were examined for histological evidence of damage to these organs. At the dosages of ET-18-$OCH_3$ or of fish oil plus ferric citrate used in this study, neither serum enzyme levels nor microscopic examination of the histological sections of liver, lungs or spleen indicated damage that could be attributed to the dietary modifications or to the ET-18-$OCH_3$. All mice appeared healthy and active, had similar food intake and had similar weight gains during the experiment. Thus, there were no detectable harmful side-effects on the host mouse because of the dietary modifications or because of the ET-18-$OCH_3$ treatment. Additionally, as there were no significant differences between groups in food consumption or weight gains, food restriction cannot account for suppression of tumour growth.

### Possible mechanisms of action leading to tumour regression

The gross measurements and the microscopic examination of histological sections of tumours revealed that there was an increase in tumour cell death because of consumption of the fish oil and ferric citrate. The result was that the mean tumour growth rate was almost nil in the mice that consumed 19% fish oil plus ferric citrate either with or without ET-18-$OCH_3$. What mechanisms related to fatty acid and iron consumption would explain tumour regression?

#### *Role of alteration in eicosanoid production*

Eicosanoids are important regulators of cell proliferation and of the immune response. It has been shown that *n*-3 fatty acids suppress the synthesis of eicosanoids in the tumour (Rose et al, 1995). However, no measures of eicosanoids were made in this study so we have no evidence for further comment.

#### *Role of lipid peroxidation*

Both cancer cells and normal cells will incorporate HPUFAs in the cellular membranes when exogenous HPUFAs are supplied in the diet. The HPUFAs in cellular membranes readily undergo spontaneous lipid peroxidation and free radicals are formed. The free radicals can react with other HPUFA chains in cellular membranes resulting in a cascade of lipid peroxidation and free radical formation. If the process is not halted, lipid peroxidation products can reach cytotoxic levels. However, normal cells do not accumulate lipid peroxidation products to the same extent as do cancer cells. This difference between normal cells and cancer cells in the accumulation of lipid peroxidation products is attributed to: compartmentalization and protection from oxygen free radicals; the presence of effective cellular antioxidants; and the effective removal and neutralization of peroxidized lipids by the normal cells (Horrobin, 1994). In support of the role of increased lipid peroxidation, it has been reported that there is less regression of tumours when lipid peroxidation was suppressed by addition of antioxidants to a diet containing fish oil and ferric citrate (Gonzalez et al, 1991). It is therefore hypothesized that these fundamental differences in the ability to handle oxidative stress between normal and cancer cells can be exploited to selectively kill the cancer cells with little or no harm to the normal cells.

#### *Role of iron*

The graphic presentation of data in Figure 3 shows that increased lipid peroxidation (as measured by TBARSs) is generally negatively correlated with tumour growth. However, the data presented in Figure 3 make clear that tumour growth is not solely dependent on the accumulation of lipid peroxidation products as measured by the concentration of TBARS. The tumour growth rate in the group of mice that consumed fish oil and ferric citrate is suppressed much more than would be expected from the increase in TBARS and in fact is outside the 95% confidence interval for the regression of TBARS against growth rate for the other three groups. This indicates that addition of dietary iron, in the form of ferric citrate, makes an additional contribution to suppression of the tumour growth rate. Apparently, the mechanism by which iron works to cause tumour regression is in addition to iron's ability to enhance the generation of lipid peroxidation products (as measured by TBARS). It is important to recognize that, in addition to malondialdehyde, 4-hydroxynonenal and 4-hydroxyhexenal may also be formed during lipid peroxidation and the formation of these two alkenals might also be enhanced in the presence of iron. These alkenals are detected by the TBARS reaction; however, they may be present and are cytotoxic at micromolar concentrations (Esterbauer et al, 1991) and would constitute but a small fraction of the TBARS. An increase in the localized concentration of these alkenals could in this way increase cell death without a measurable increase in the TBARS level.

The suggestion of another mechanism of how iron worked to make an additional contribution to suppression of carcinoma growth stems from the fact that iron supplementation enhanced the cytotoxic index without lowering the mitotic index. The increased cell death could be because of iron enhanced free radical damage to proteins or to DNA, which would not be measured by TBARS.

Yet another possible mechanism for the effect of iron on tumour cell death could be an increased uptake of iron into the cancer cells. Breast cancer cells often have higher numbers of transferrin receptors than normal breast epithelial cells (Inoue et al, 1993). Thus, the cancer cells may have sequestered a higher level of iron, resulting in increased damage from the iron even though the mean serum iron was not significantly increased in the group that received supplemental iron. Future research is needed to: elucidate the mechanism(s) by which iron supplementation, in this system, selectively facilitated cancer cell death without suppression of mitotic activity; determine if other types, amounts and preparations of PUFA will be as effective as Menhaden fish oil in tumour suppression; and determine any long-term side-effects of the therapeutic approach. The marked tumour suppressive results obtained in this study clearly warrant further research on this novel and effective approach to breast cancer therapy. The results of this research suggest that a cancer therapy protocol incorporating omega-3 fatty acids and iron might be an effective cancer therapy for humans.

### ACKNOWLEDGEMENTS

ET-18-$OCH_3$ was a gift from Medmak Pharma, Germany. Funding was provided by grants from the American Institute for Cancer Research and from the Veterans Administration. Mrs Willie L Grant is thanked for technical assistance.

### REFERENCES

Begin ME, Ells G, Das UN and Horrobin DF (1986) Differential killing of human carcinoma cells supplemented with *n-3* and *n-6* polyunsaturated fatty acids. *J Natl Cancer Inst* **77**: 1053–1062

Begin ME, Ells, G and Horrobin DF (1988) Polyunsaturated fatty acid-induced cytotoxicity against tumor cells and its relationship to lipid peroxidation. *J Natl Cancer Inst* **80**: 188–194

© Cancer Research Campaign 1997 

Borgeson CE, Pardini L, Pardini RS and Reitz RC (1989) Effects of dietary fish oil on human mammary carcinoma and on lipid-metabolizing enzymes. *Lipids* **24**: 290–295

Bradford MM (1976) A rapid and sensitive method for the quantitation of microgram quantities of protein utilizing the principle of protein-dye binding. *Anal Biochem* **72**: 248–254

Canuto RA, Muzio G, Biocca ME and Dianzani MU (1991) Lipid peroxidation in rat AH-130 hepatoma cells enriched in vitro with arachidonic acid. *Cancer Res* **51**: 4603–4608

Corrocher R, Casaril M, Bellisola G, Gabriella GB, Nicoli N, Guidi GC and De Sandre G (1986) Severe impairment of antioxidant system in human hepatoma. *Cancer* **58**: 1658–1662

Davidson I and Henry JB (1974) *Clinical Diagnosis by Laboratory Methods*. WB Saunders: Philadelphia

Esterbauer H, Schaur RJ and Zollner H (1991) Chemistry and biochemistry of 4-hydroxynonenal, malondialdehyde and related aldehydes. *Free Rad Biol Med* **11**: 81–128

Gonzalez MJ, Schemmel RA, Gray JI, Dugan LJ, Sheffield LG and Welsch CW (1991) Effect of dietary fat on growth of MCF-7 and MDA-MB231 human breast carcinomas in athymic nude mice: Relationship between carcinoma growth and lipid peroxidation product levels. *Carcinogenesis* **12**: 1231–1235

Gonzalez MJ, Gray JI, Schemmel RA, Dugan LJ and Welsch CW (1992) Lipid peroxidation products are elevated in fish oil diets even in the presence of added antioxidants. *J Nutr* **122**: 2190–2195

Gonzalez MJ, Schemmel RA, Dugan L, Gray JI and Welsch CW (1993) Dietary fish oil inhibits human breast carcinoma growth: a function of increased lipid peroxidation. *Lipids* **28**: 827–832

Grammatikos SI, Subbaiah PV, Victor TA and Miller WM (1994) *n-3* and *n-6* fatty acid processing and growth effects in neoplastic and non-cancerous human mammary epithelial cell lines. *Br J Cancer* **70(2)**: 219–227

Horrobin DF (1994) Unsaturated lipids and cancer. In *New Approaches to Cancer Treatment: Unsaturated Lipids and Photodynamic Therapy*, Horrobin DF (ed.), pp. 3–29 Churchill Livingston: London

Inoue T, Cavanaugh PG, Steck PA, Brunner N and Nicholson GL (1993) Differences in transferrin response and numbers of transferrin receptors in rat and human mammary carcinoma lines of different metastatic potentials. *J Cell Physiol* **156**: 212–217

Leder GH, Fiebig HH, Wallbrecher E, Winterhalter BR and Lohr GW (1987) In vitro and in vivo cytotoxicity of the alkyl lysophospholipid ET-18-$OCH_3$ and the thioether lipid BM 41.440. *Lipids* **22**: 958–961

McConkey DJ and Orrenius S (1994) Signal transduction pathways to apoptosis. *Trends Cell Biol* **4**: 370–375

Maehle L, Eilertsen E, Mollerup S, Schonberg S, Krokan HE and Haugen A (1995) Effects of *n-3* fatty acids during neoplastic progression and comparison of in vitro and in vivo sensitivity of two human tumour cell lines. *Br J Cancer* **71**: 691–696

Masotti L, Casali E and Galeotti T (1988) Lipid peroxidation in tumor cells. *Free Rad Biol Med* **4**: 377–386

Moyer MP and Aust JB (1984) Human colon cells: culture and in vitro transformation. *Science* **224**: 1445–1447

Padma M and Das UN (1996) Effect of cis-unsaturated fatty acids on cellular oxidant stress in macrophage tumor (AK-5) cells in vitro. *Cancer Lett* **109**: 63–75

Pritchard GA, Jones DL and Mansel RE (1989) Lipids in breast carcinogenesis. *Br J Surg* **76**: 1069–1073

Rose DP, Connolly JM, Rayburn J and Coleman M (1995) Influence of diets containing eicosapentaenoic or docosahexaenoic acid on growth and metastasis of breast cancer cells. *J Natl Cancer Inst* **87**: 587–592

Wagner BA, Buettner GR and Burns CP (1992) Membrane peroxidative damage enhancement by the ether lipid class of antineoplastic agents. *Cancer Res* **52**: 6045–6051

© Cancer Research Campaign 1997


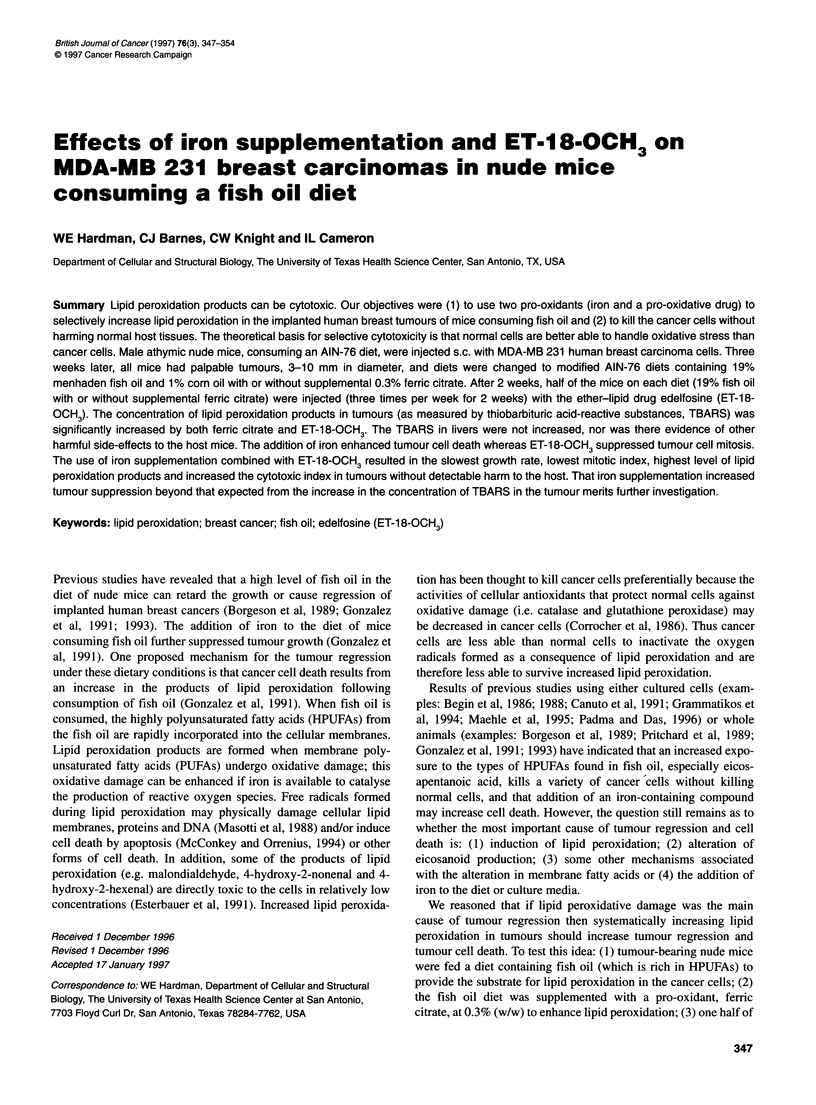

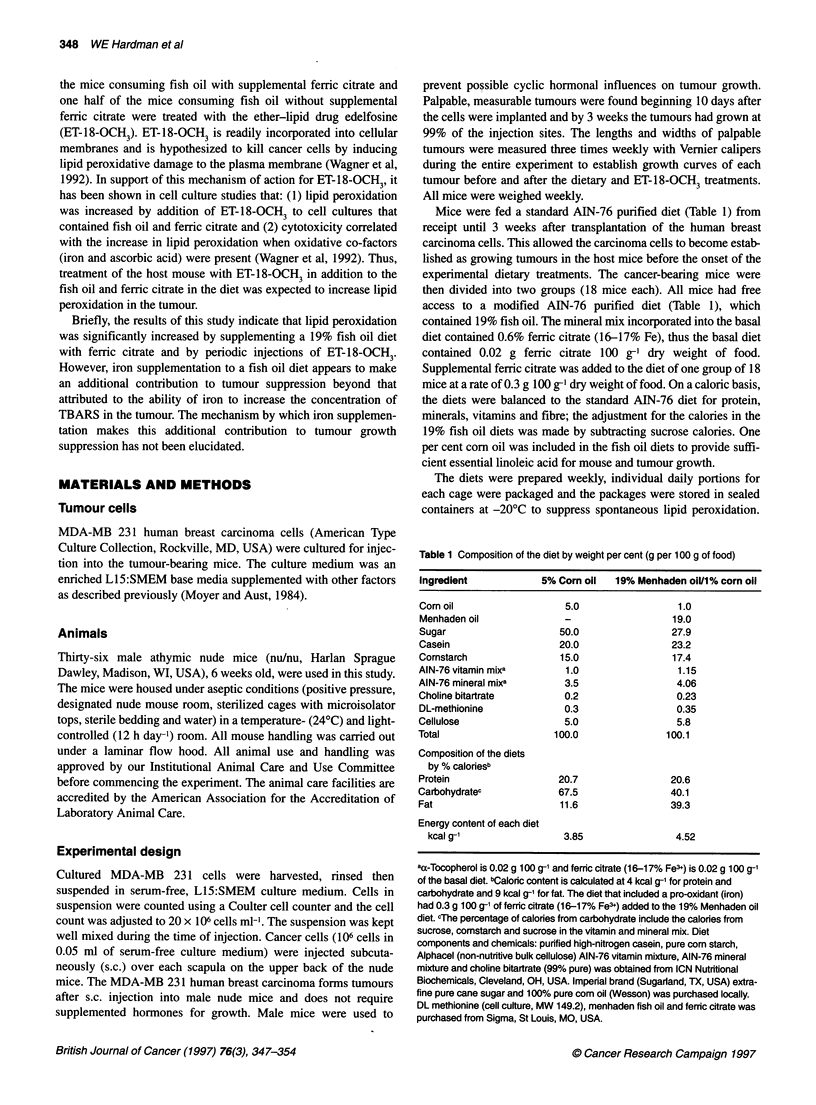

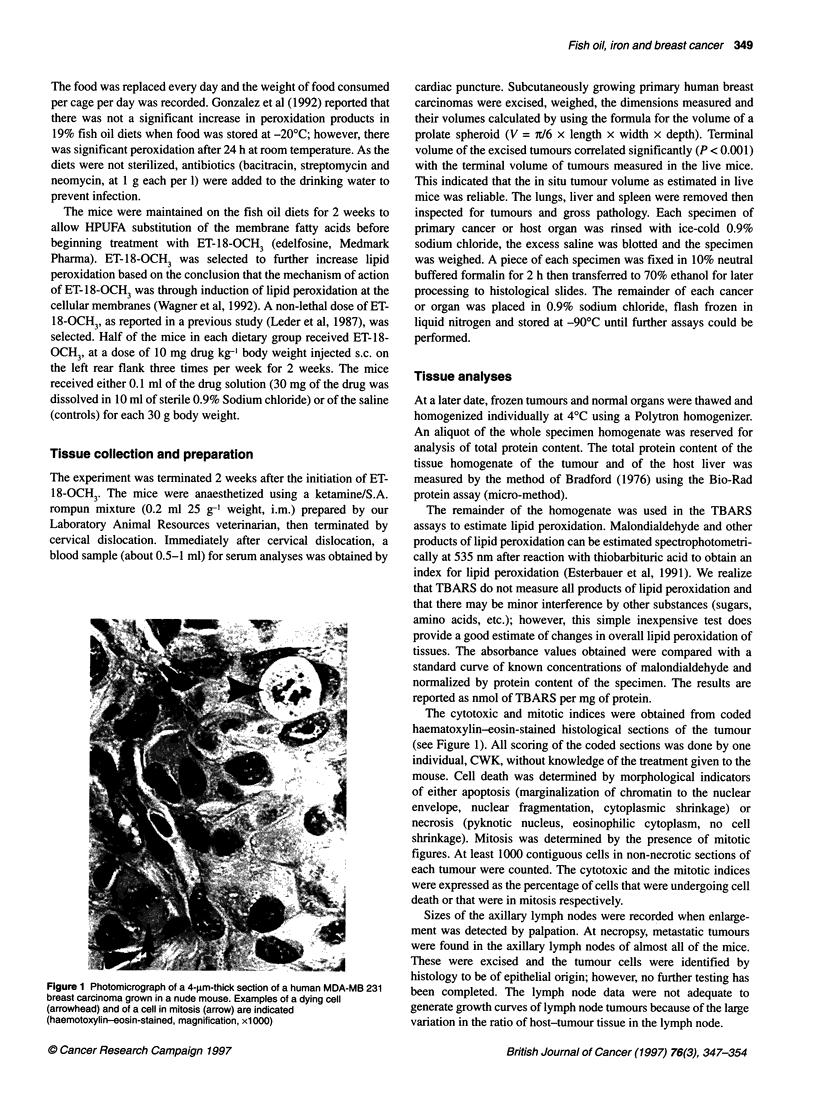

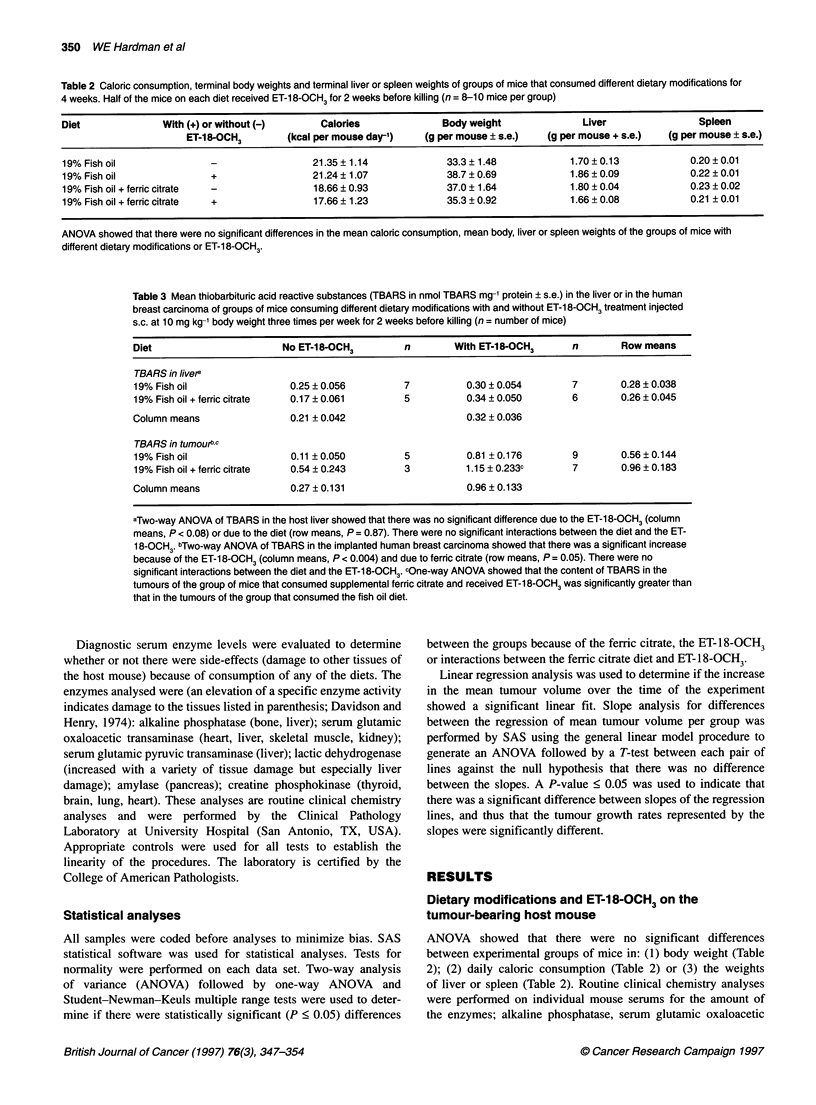

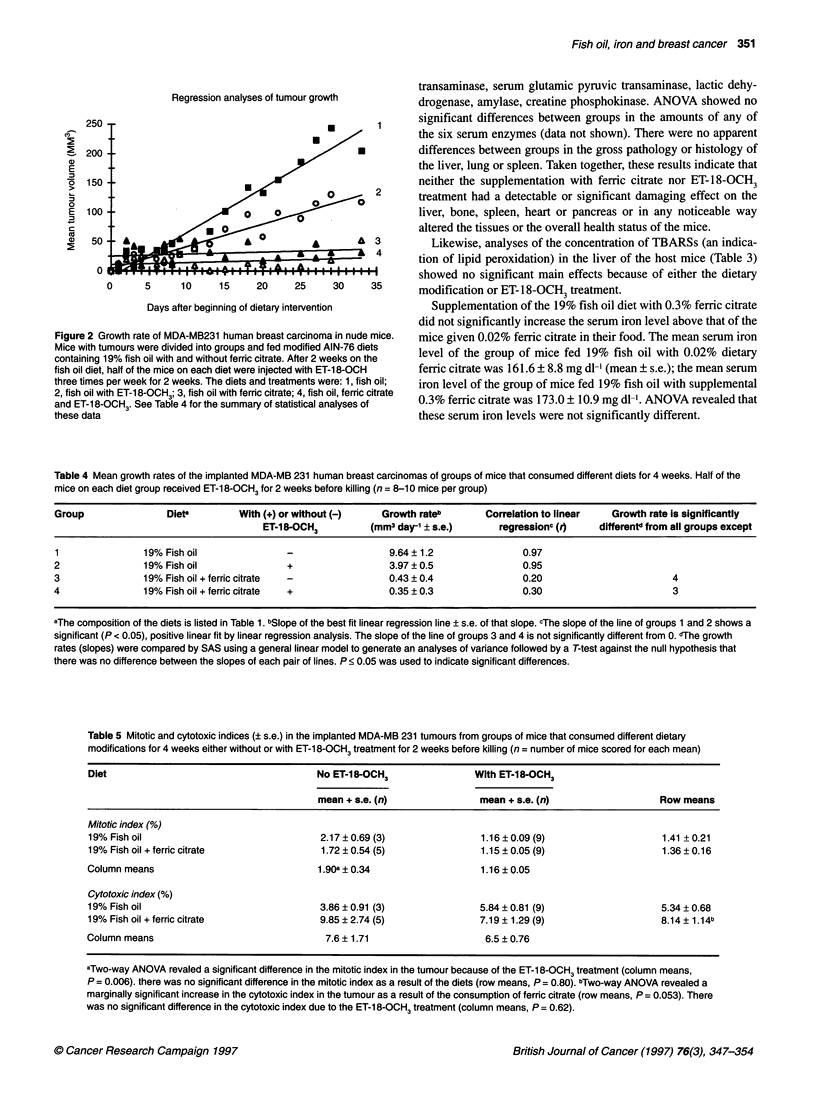

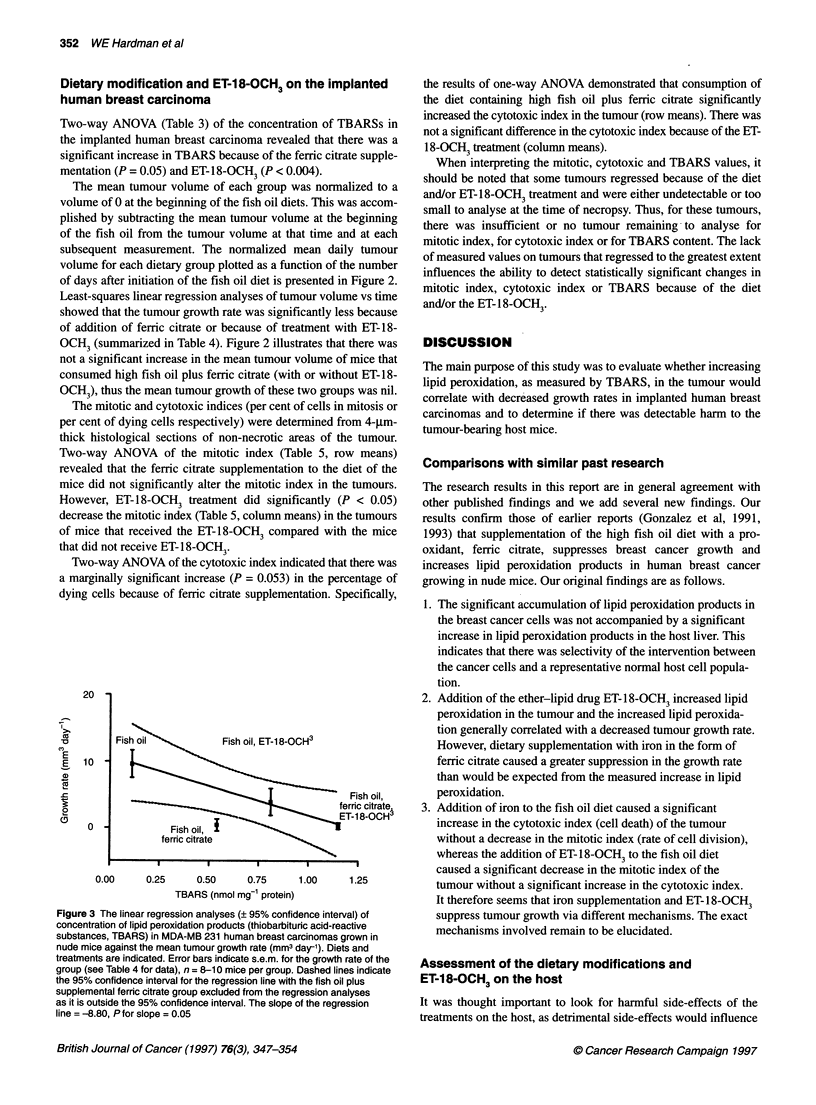

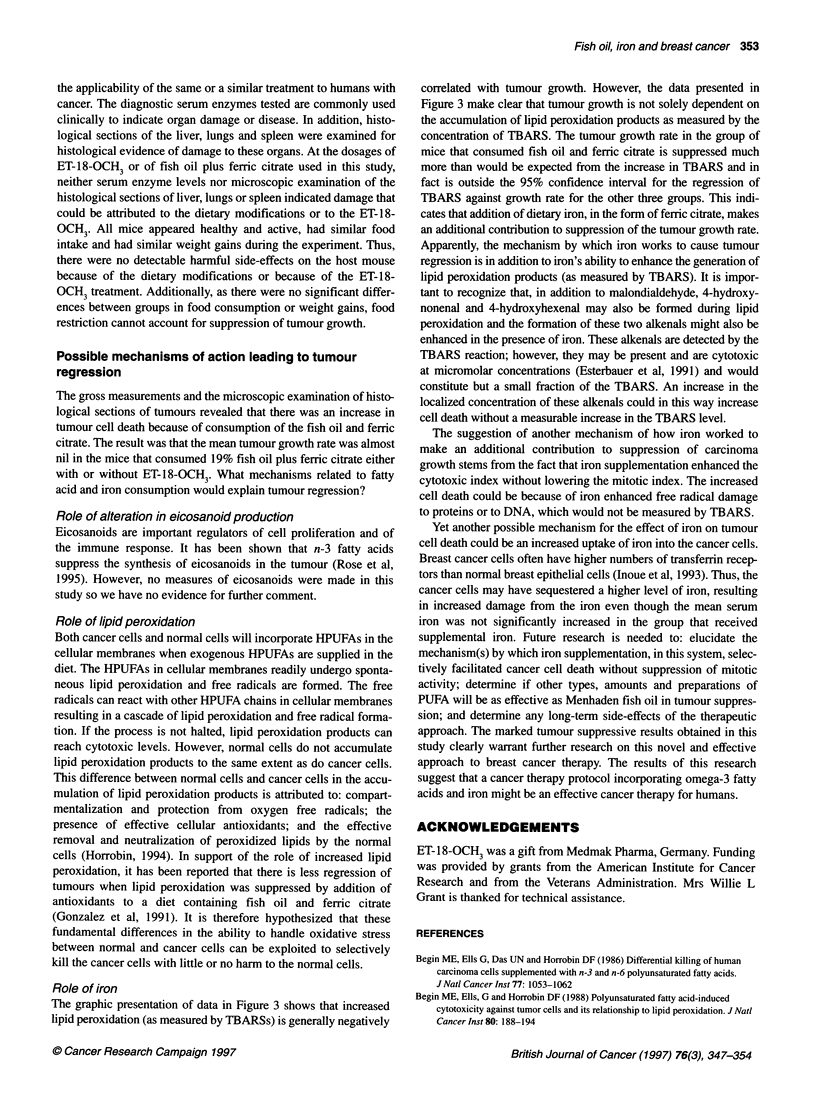

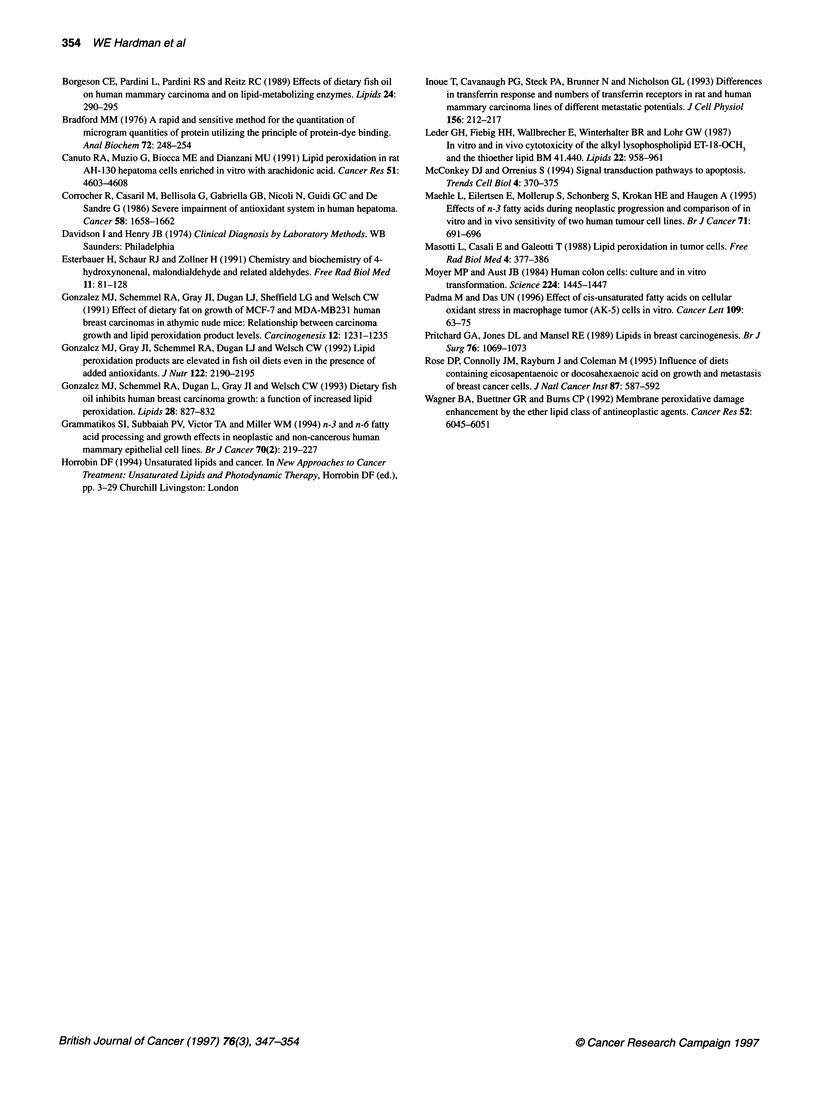


## References

[OCR_00845] Borgeson C. E., Pardini L., Pardini R. S., Reitz R. C. (1989). Effects of dietary fish oil on human mammary carcinoma and on lipid-metabolizing enzymes.. Lipids.

[OCR_00850] Bradford M. M. (1976). A rapid and sensitive method for the quantitation of microgram quantities of protein utilizing the principle of protein-dye binding.. Anal Biochem.

[OCR_00831] Bégin M. E., Ells G., Das U. N., Horrobin D. F. (1986). Differential killing of human carcinoma cells supplemented with n-3 and n-6 polyunsaturated fatty acids.. J Natl Cancer Inst.

[OCR_00836] Bégin M. E., Ells G., Horrobin D. F. (1988). Polyunsaturated fatty acid-induced cytotoxicity against tumor cells and its relationship to lipid peroxidation.. J Natl Cancer Inst.

[OCR_00855] Canuto R. A., Muzio G., Biocca M. E., Dianzani M. U. (1991). Lipid peroxidation in rat AH-130 hepatoma cells enriched in vitro with arachidonic acid.. Cancer Res.

[OCR_00860] Corrocher R., Casaril M., Bellisola G., Gabrielli G. B., Nicoli N., Guidi G. C., De Sandre G. (1986). Severe impairment of antioxidant system in human hepatoma.. Cancer.

[OCR_00869] Esterbauer H., Schaur R. J., Zollner H. (1991). Chemistry and biochemistry of 4-hydroxynonenal, malonaldehyde and related aldehydes.. Free Radic Biol Med.

[OCR_00880] Gonzalez M. J., Gray J. I., Schemmel R. A., Dugan L., Welsch C. W. (1992). Lipid peroxidation products are elevated in fish oil diets even in the presence of added antioxidants.. J Nutr.

[OCR_00885] Gonzalez M. J., Schemmel R. A., Dugan L., Gray J. I., Welsch C. W. (1993). Dietary fish oil inhibits human breast carcinoma growth: a function of increased lipid peroxidation.. Lipids.

[OCR_00874] Gonzalez M. J., Schemmel R. A., Gray J. I., Dugan L., Sheffield L. G., Welsch C. W. (1991). Effect of dietary fat on growth of MCF-7 and MDA-MB231 human breast carcinomas in athymic nude mice: relationship between carcinoma growth and lipid peroxidation product levels.. Carcinogenesis.

[OCR_00890] Grammatikos S. I., Subbaiah P. V., Victor T. A., Miller W. M. (1994). n-3 and n-6 fatty acid processing and growth effects in neoplastic and non-cancerous human mammary epithelial cell lines.. Br J Cancer.

[OCR_00900] Inoue T., Cavanaugh P. G., Steck P. A., Brünner N., Nicolson G. L. (1993). Differences in transferrin response and numbers of transferrin receptors in rat and human mammary carcinoma lines of different metastatic potentials.. J Cell Physiol.

[OCR_00906] Leder G. H., Fiebig H. H., Wallbrecher E., Winterhalter B. R., Löhr G. W. (1987). In vitro and in vivo cytotoxicity of alkyl lysophospholipid ET-18-OCH3 and thioether lipid BM 41.440.. Lipids.

[OCR_00915] Maehle L., Eilertsen E., Mollerup S., Schønberg S., Krokan H. E., Haugen A. (1995). Effects of n-3 fatty acids during neoplastic progression and comparison of in vitro and in vivo sensitivity of two human tumour cell lines.. Br J Cancer.

[OCR_00921] Masotti L., Casali E., Galeotti T. (1988). Lipid peroxidation in tumour cells.. Free Radic Biol Med.

[OCR_00911] McConkey D. J., Orrenius S. (1994). Signal transduction pathways to apoptosis.. Trends Cell Biol.

[OCR_00925] Moyer M. P., Aust J. B. (1984). Human colon cells: culture and in vitro transformation.. Science.

[OCR_00929] Padma M., Das U. N. (1996). Effect of cis-unsaturated fatty acids on cellular oxidant stress in macrophage tumor (AK-5) cells in vitro.. Cancer Lett.

[OCR_00934] Pritchard G. A., Jones D. L., Mansel R. E. (1989). Lipids in breast carcinogenesis.. Br J Surg.

[OCR_00938] Rose D. P., Connolly J. M., Rayburn J., Coleman M. (1995). Influence of diets containing eicosapentaenoic or docosahexaenoic acid on growth and metastasis of breast cancer cells in nude mice.. J Natl Cancer Inst.

[OCR_00943] Wagner B. A., Buettner G. R., Burns C. P. (1992). Membrane peroxidative damage enhancement by the ether lipid class of antineoplastic agents.. Cancer Res.

